# Intestinal Perforation in Obstructed Umbilical Hernia due to Wedged Plum Seed

**DOI:** 10.21699/ajcr.v7i3.439

**Published:** 2016-06-15

**Authors:** Rahul Gupta, Vikram Singh Mujalde, Shilpi Gupta, Pradeep Kumar Gupta, Anu Bhandari, Praveen Mathur

**Affiliations:** 1 Department of Paediatric Surgery, SMS Medical College Jaipur, Rajasthan, India; 2 Department of Radiodiagnosis, SMS Medical College Jaipur, Rajasthan, India

**Keywords:** Intestinal perforation, Plum seed, Obstructed hernia, Umbilical hernia

## Abstract

The foreign body ingestion is a rare cause of gastrointestinal perforation in children and is typically seen with sharp foreign bodies or button batteries. Herein, we report an 11-month old male baby who presented with obstructed umbilical hernia. Abdominal radiograph showed dilated small bowel loops, while ultrasonography and CT scan suggested presence of a foreign body. Laparotomy revealed obstructed umbilical hernia with a plum seed being stuck in the terminal ileum causing intestinal perforation. Resection and anastomosis of intestine was performed.

## INTRODUCTION

The foreign body ingestion is a relatively common clinical problem in children. Most of them pass through the gastrointestinal tract spontaneously.[1,2] However, esophagus and small bowel are the most commonly involved sites of perforation and is typically seen with sharp foreign bodies or button batteries ingestion.[3,4] The presentation of foreign body as incarcerated umbilical hernia has been described in literature.[5] Herein, we report a case of perforation in obstructed umbilical hernia due to plum seed wedged in the terminal ileum.

## CASE REPORT

An 11-month old male infant presented with bilious vomiting, abdominal distension and swelling in the umbilical region along with constipation for three days. Umbilical swelling was previously reducible. On examination, the patient was hemodynamically stable with mild dehydration, respiratory rate of 33/min, and pulse rate of 120/min. On physical examination, abdominal distention was present along with tender lump in the umbilical region suggestive of obstructed umbilical hernia. Laboratory investigations revealed total leucocyte count-20,600 mm3, hemoglobin-10.1 gm/dl, with normal renal and liver functions. Abdominal x-ray showed dilated small bowel loops (fig.1); ultrasonography (USG) suggested 22 mm umbilical hernial defect with herniated bowel loops, small septate collection, along with a hyperechoic lesion with distal acoustic shadows in the umbilical region. CT scan demonstrated a 2.7 cm x 1 cm x 1 cm hyperdense lesion as content of the hernia (fig.1). Operation revealed obstructed umbilical hernia with terminal ileum (1-2 cms from ileo-cecal region) as its content (fig. 2). Non-fixation of caecum and ascending colon was present. A plum seed which was wedged in the terminal ileum was seen to be coming out from the perforation in the bowel (fig. 2). The seed was lying transversely resulting in two perforations in terminal ileum. The ileo-cecal region was markedly congested and edematous. Primary closure of gut was not feasible (fig. 2). Resection of the ileo-cecum with ileo-ascending colon anastomosis was performed. Postoperative period was unremarkable, except for minor wound infection. Patient was started on oral feeds on the fifth postoperative day and remained well at follow up after discharge.

**Figure:**
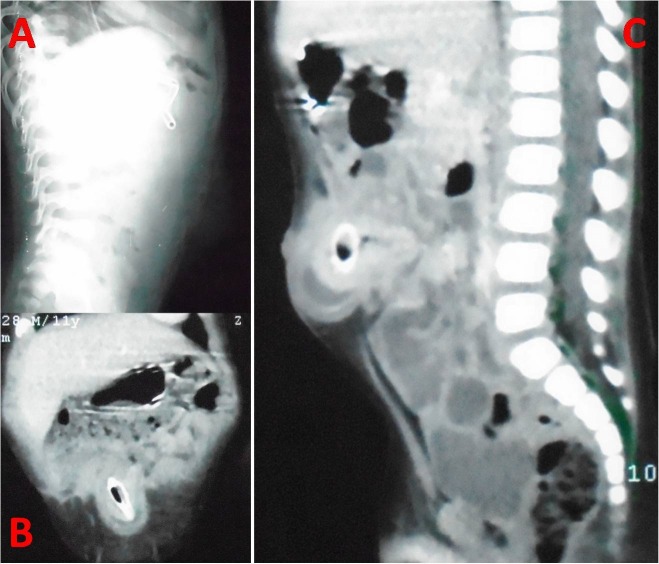
Figure 1:Preoperative radiograph showing a soft tissue shadow suggesting umbilical hernia (lateral view, A); Abdominal computed tomography scan images showing a foreign body as content of the umbilical hernia (B, C).

**Figure:**
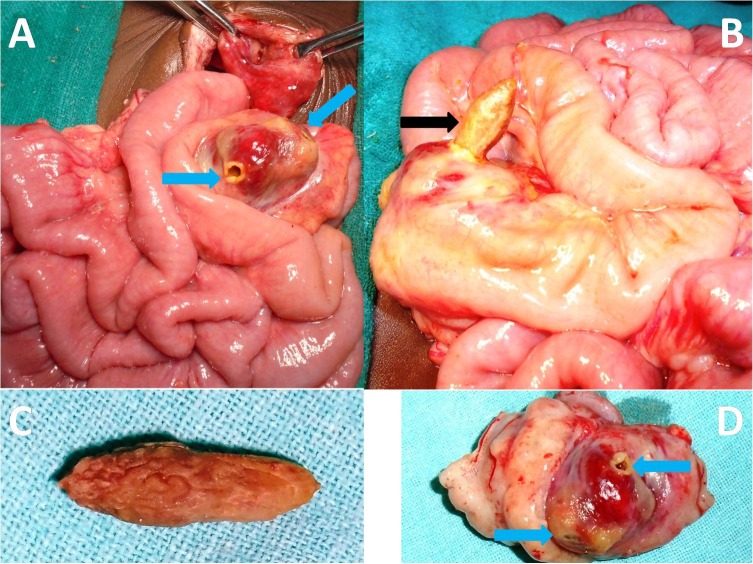
Figure 2:Per-operative photographs showing umbilical hernial sac, terminal ileum (1-2 cms from ileo-cecal region) incarcerated in umbilical hernia is seen with two perforations (diagonally opposite to each other as shown by blue arrows) due to wedged plum seed (A); Plum seed (black arrow) coming out through perforation (B); Plum seed (C) and resected specimen (D) with perforations (blue arrow).

## DISCUSSION

Most of the ingested foreign bodies pass through the gastrointestinal tract (GIT) spontaneously. Few stuck (less than 1%) in the anatomical transition regions (at the narrowing and angulations) of the GIT which include the upper, middle and lower esophagus, pylorus, ileocecal valve and rectosigmoid colon. Esophagus and small bowel are the most commonly locations involved.[3-5] Foreign bodies with sharp edges lead to serious complications (15%-35%) like intestinal obstruction, perforation and erosion into adjacent organs.[6] Apart from plum seed, apricots, persimmons, peanuts and nectarine have been reported to cause perforation.[7] It appears that in our case, presence of seed itself led to irreducibility of the previously reducible umbilical hernia and also sharp ends of the seed, in addition to attempt by peristalsis led to traumatic perforation in bowel inside the obstructed umbilical hernia.

Perforations of the appendix and Meckel’s diverticulum due to a seed ingestion have also been described.[8,9] A rare case of incarcerated inguinal hernia due to ingested foreign body has also been reported.[10] Foreign body ingestion presenting as obstructed umbilical hernia is extremely rare.[5] Senior author (PM) encountered a similar case of obstructed umbilical hernia due to ingestion of turmeric stem in a 5-year-old boy.

## Footnotes

**Source of Support:** Nil

**Conflict of Interest:** None declared

